# Vertical distribution of the relic species *Eurytemora lacustris* (Copepoda, Calanoida) in stratified mesotrophic lakes

**DOI:** 10.2478/s11756-018-0138-y

**Published:** 2018-10-26

**Authors:** Maciej Karpowicz, Krystyna Kalinowska

**Affiliations:** 1 Institute of Biology, Department of Hydrobiology, University of Białystok, Ciołkowskiego 1J, 15-245 Białystok, Poland; 2 Department of Lake Fisheries, Inland Fisheries Institute in Olsztyn, Rajska 2, 11-500 Giżycko, Poland

**Keywords:** *Eurytemora lacustris*, Vertical distribution, Environmental factors, Food resources, Epibionts

## Abstract

The aim of this study was to determine factors affecting the vertical distribution of *Eurytemora lacustris* in mesotrophic lakes (Wigry, Hańcza, Szurpiły; north-eastern Poland) during the summer stagnation. *Eurytemora lacustris* was found in all of the studied lakes, with the highest abundance (8 ind. L^−1^) in Lake Wigry. In Lake Szurpiły, *E. lacustris* has never been recorded before. The results of this study revealed that *E. lacustris* was most numerous in thermocline zones, suggesting that this species could temporarily tolerate warmer water and lower oxygen concentrations due to better food resources. During the study, it was found that a large part of the *E. lacustris* population had epibiont ciliates, in contrast to other species of zooplankton that did not have any epibionts. The improvement in the water quality of many deep lakes could lead to an increase in the abundance of *E. lacustris*. However, epibiont ciliates may be a threat for this species and may play a substantial role in determining the production, distribution, and community dynamics of *E. lacustris*.

## Introduction

*Eurytemora lacustris* (Poppe, 1887) is considered a ‘classical’ glacial relict species in central Europe (Maier et al. 2011), while the inclusion of this species in the relict element in Norway and Sweden has been discussed (Kinsten 2012; Spikkeland et al. 2016). From the zoogeographical point of view, *E. lacustris* is a typical so-called Baltic species, with a distribution related to the Ancylus stage of the post-glacial Baltic Sea development (Spikkeland et al. 2016). According to Thienemann (1950), *E. lacustris* is a cold-adapted calanoid copepod which spread over parts of Europe by glacier lakes during the last ice age, about 8500 years ago. The glaciers “inoculated” the species into the lake basins that had been created in the meantime. During the post-glacial period, when the temperatures gradually increased, *E. lacustris* was able to survive by retreating to the cold water layers of thermally stratified lakes. *Eurytemora lacustris* occurs exclusively in freshwater ecosystems located in the area from the Boreal highlands of northern Norway to the Black Sea and from the central European lowlands to the eastern parts of the Caspian region (Illies 1978; Spikkeland et al. 2016). However, this species has become rare and endangered due to eutrophication and global change (Maier et al. 2011). As a result of anthropogenic increases in external nutrient loading in lakes of central Europe over the past few decades (Gulati and Van Donk 2002), *E. lacustris* disappeared from many lakes, as it is a species which is very sensitive to environmental deterioration (Arbačiauskas and Kalytytė 2010). European countries have made great efforts to improve the ecological quality of lakes by reducing external nutrient loading (Sas 1989) or additional restoration measures such as lake biomanipulation, or both (e.g. Benndorf 1990, 2005; Kornijów et al. 1990; Hansson et al. 1998; Mehner et al. 2002). As a consequence, re-oligotrophication is a well-documented phenomenon in many lakes (Jeppesen et al. 2005), and *E. lacustris* is more common nowadays in deep lakes in central Europe. The high habitat requirements make *E. lacustris* an excellent indicator of the effectiveness of deep lake restoration. This species is also a very good indicator of the low trophic level and good ecological status of lakes (Karabin 1985; Ejsmont-Karabin and Karabin 2013; Ochocka and Pasztaleniec 2016).

*Eurytemora lacustris* requires cold and well-oxygenated waters; thus, it was found in freshwater lakes, which were mostly oligotrophic, deep (max depth > 30 m), and oxygenated (>1 mg O_2_ L^−1^), with cold hypolimnion (usually <10 °C, with a threshold at 17 °C) (Rylov 1935; Patalas and Patalas 1966; Kasprzak et al. 2005). Based on the literature data, the combined effects of temperature and oxygen concentration in deep waters seem to be fundamental factors influencing the occurrence of *E. lacustris*. Some studies indicated that *E. lacustris* exhibits a distinct diurnal vertical migration (Ruttner 1905; Adlerówna 1929), but the migration amplitude clearly decreased during the summer and was closely related to the light intensity (Kasprzak et al. 2005). Other studies reported small differences between day and night in the vertical distribution of this species, e.g. the copepodites and adults were slightly higher at night than during the day (Weiler et al. 2003). The vertical distributions of *E. lacustris* vary seasonally. In the summer, this species is mainly restricted to the cold, lower water layers, while in the winter and during periods of mixis, it prefers the upper water layers or is uniformly distributed throughout the water column (Weiler et al. 2003). However, the factors which affect the vertical distribution of this species during the summer stagnation are unclear.

The aim of this study was to determine abiotic (temperature, oxygen) and biotic (phytoplankton) factors affecting the vertical distribution of *E. lacustris* in three mesotrophic lakes (Wigry, Hańcza, Szurpiły) in north-eastern Poland. During the study, it was found that a large part of the *E. lacustris* population had epibiont ciliates in contrast to other species of zooplankton that did not have any epibionts. Therefore, an additional goal of this study was to assess the infection parameters of *E. lacustris* populations, such as the prevalence and average intensity of the epibiont suctorian ciliates.

## Material and methods

The study was conducted in three mesotrophic lakes in NE Poland: Lake Hańcza (the deepest lake in central and Eastern Europe, with a maximum depth of 108.5 m), Lake Szurpiły (with a maximum depth of 40.0 m) and Lake Wigry (with a maximum depth of 74.0 m). In Lake Wigry, samples were collected from two basins (the North Basin with a maximum depth exceeding 65.0 m and the Central Basin with a maximum depth of 73.0 m). Morphometric and trophic parameters of the studied lakes are shown in Table 1. The trophic state index (TSI) of lakes was calculated from Secchi disc visibility (Z_SD_) and chlorophyll *a* concentrations according to Carlson (1977). The study was conducted during the peak of the summer stagnation in July 2015 (Lakes Hańcza and Szurpiły) and July 2016 (Lake Wigry) between 11:00 and 13:00. The zooplankton samples (20 L) were taken every meter from the surface to the upper hypolimnion (0–11 m) by the 5 L Limnos sampler. Additionally, one sample was taken from the centre of the hypolimnion. Temperature, oxygen concentration, and phytoplankton communities were measured every metre from the surface to a depth of 30 m. The temperature and dissolved oxygen concentrations were determined using an HQ40D Multi Meter (Hach-Lange GmbH). The phytoplankton (total chlorophyll *a* and algal classes) was measured in situ by the submersible spectrofluorometer (FluoroProbe, bbe-Moldaenke), using differences between fluorescence excitation spectra. Changes in chlorophyll *a* emission allow for the fluorometric estimation of algal classes based on differences in species and class-dependent peripheral antenna pigments (Beutler et al. 2002). The FluoroProbe identifies the four phytoplankton classes: green algae (Chlorophyta and Euglenophyta), cyanobacteria (phycocyanin-rich cyanobacteria), diatoms (Heterokontophyta, Haptophyta, and Dinophyta) and cryptophytes (Cryptophyta and the phycoerythrin-rich cyanobacteria).

**Table 1:** Morphometric and trophic characteristics of the studied lakes

	Hańcza	Wigry	Szurpiły
Latitude	54°15.9′	54°02.4’	54°14.3′
Longitude	22°51.1′	23°07.1’	22°53.5′
Area (ha)	311.4	2118.3	80.9
Max depth (m)	108.5	74.0	40.0
Mean depth (m)	38.7	15.4	10.0
Length of coastline (m)	11,750	72,225	7000
Shoreline development	1.88	4.43	2.19
Thermocline depth (m)	8–10	8–10	6–9
Z_SD_ (m)	4.2	3.2	2.9
Chl *a* (μg L^−1^)	3.0	6.3	5.7
TSI	40.3	45.9	46.1
Trophic status	mesotrophy	mesotrophy	mesotrophy

*Z*_*SD*_, Secchi disc visibility; *Chl a*, chlorophyll *a*; *TSI*, trophic state index

**Fig. 1:**
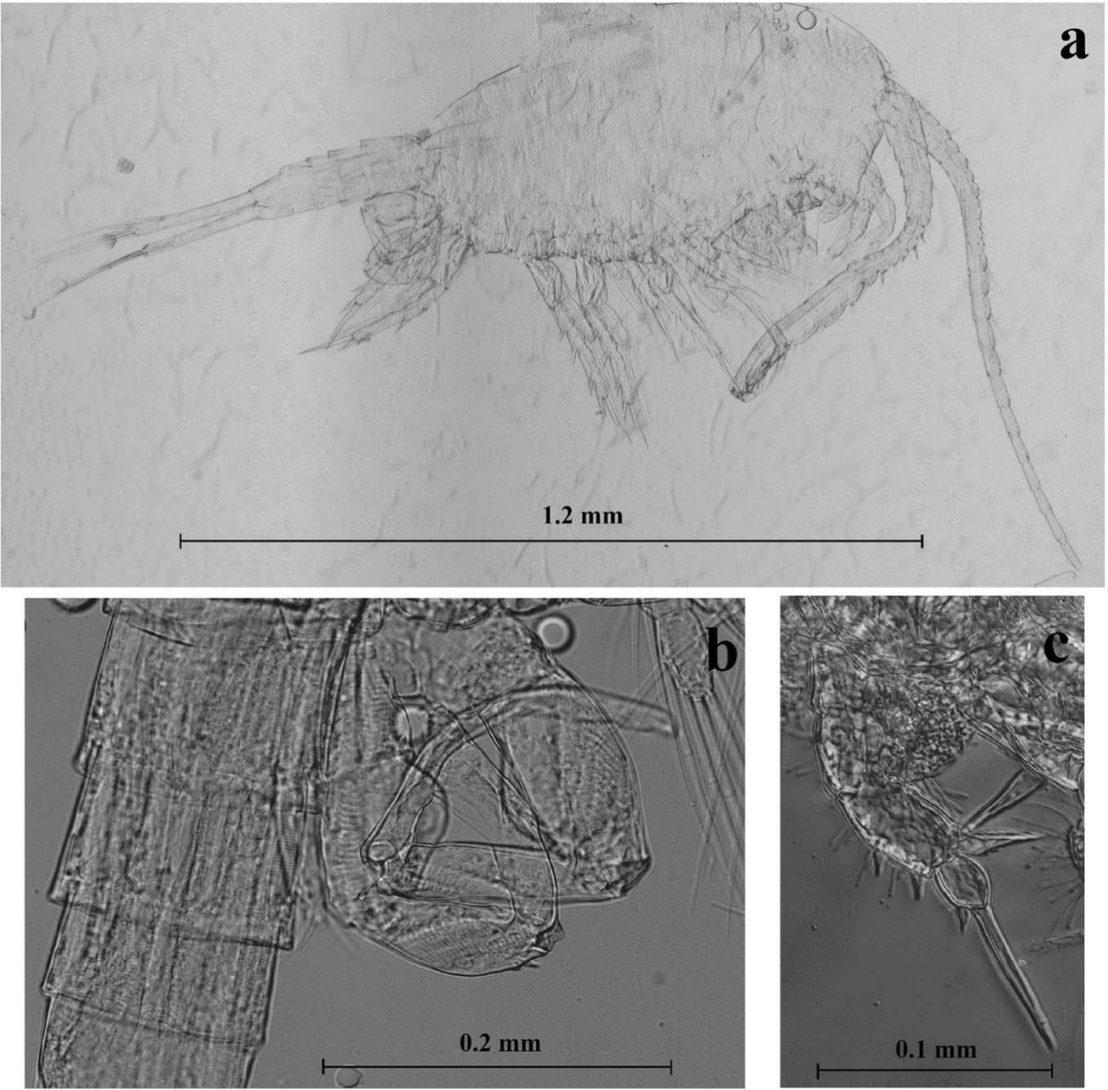
Photographs of *Eurytemora lacustris* from Lake Wigry. **a** Male, habitus. **b** The fifth pair of legs (P5) of the male. **c** The fifth pair of legs (P5) of the female

The calanoid copepod *E. lacustris* can be easily distinguished by the long furcal rami (Fig. 1a) and the shape of the fifth leg of males (Fig. 1b) and females (Fig. 1c). Other characteristic features are the 24-segmented antennule (A1) and the lack of “wings” on the 5th thoracic segment (Einsle 1993). Biomass was calculated based on the mean length using the following length-weight relationship (Błędzki and Rybak 2016):

$$ \mathrm{W}=7.9\times {10}^{-7}\ {\mathrm{L}}^{2.33} $$

W: individual body dry weight [μg]
L: body size [μm]

Species identification of epibiont ciliates was based on Foissner et al. (1995). The biomass of epibionts was calculated from measurements of cell dimensions and simple geometric shapes and then multiplied by a factor of 1.4 (Müller and Geller 1993). Quantifying epibiont numbers in *E. lacustris* populations includes the measurement of host sample size (N), prevalence (%), and mean epibiont intensity (Rózsa et al. 2000). Only adult specimens were used for analysis.

The analysis of variance was used to determine which explanatory variables (temperature, oxygen, and phytoplankton) provide significant information on the vertical distribution of *E. lacustris*. Then, we used type I sum of squares analysis to find out if the three variables and their interaction provide the same amount of information to the model. Finally, the relationships between the abundance of *E. lacustris* and environmental variables were visualized by principal component analysis (PCA). Statistical analyses were performed with XLSTAT-Ecology (Addinsoft).

## Results

**Fig. 2:**
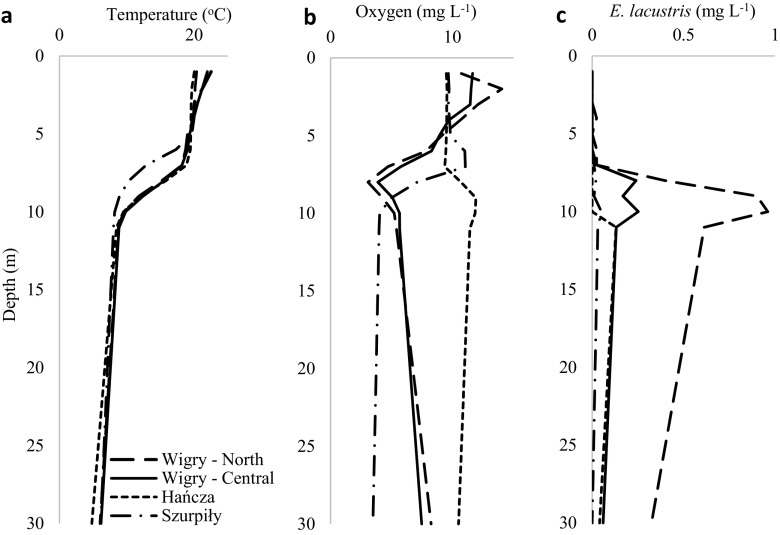
Vertical gradients of temperature (**a**), oxygen (**b**) and distribution of *Eurytemora lacustris* (**c**)

**Fig. 3:**
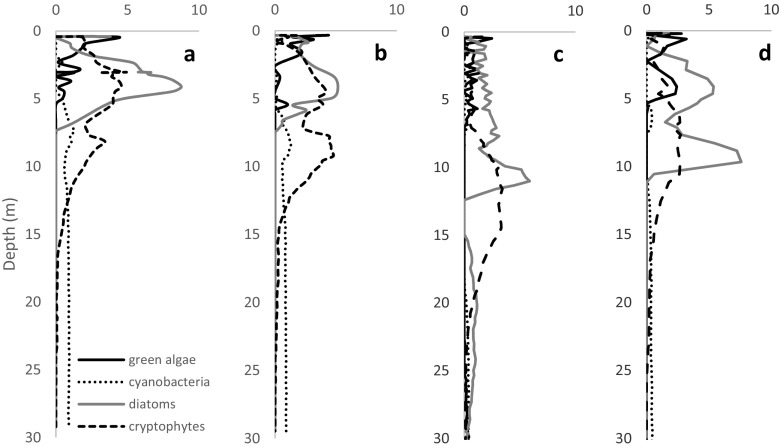
Vertical distribution of dominant phytoplankton groups (μg L^−1^) in lakes Wigry – North Basin (**a**), Wigry – Central Basin (**b**), Hańcza (**c**) and Szurpiły (**d**)

Water temperature was very similar in the studied lakes (about 20, 12 and 6–7 °C in the epi-, meta- and hypolimnion, respectively) (Fig. 2a). Thermocline developed at a depth of 8–10 m in lakes Hańcza and Wigry and at a depth of 6–9 m in Lake Szurpiły. During the study period, oxygen concentrations were almost the same in the epilimnion of all of the studied lakes (about 10 mg L^−1^). There were differences in the oxygen concentration in the thermocline between Lake Wigry (metalimnetic oxygen minimum) and Lake Hańcza (metalimnetic oxygen maximum). In lakes Hańcza and Wigry, hypolimnetic waters were quite well oxygenated, whereas the oxygen concentration was relatively low in Lake Szurpiły (Fig. 2b). In lakes Hańcza and Szurpiły, the highest concentration of algae was observed in the metalimnion, while the highest concentration in Lake Wigry was in the epilimnion (Fig. 3). The dominant groups of phytoplankton in the thermocline were cryptophytes in Lake Wigry (Fig. 3a, b), and diatoms with cryptophytes in lakes Hańcza and Szurpiły (Fig. 3c, d).

The lakes were characterised by a large number of zooplankton species, among which *Daphnia cucullata* (Sars, 1862) dominated. The genus *Daphnia* was also represented by *Daphnia cristata* G.O. Sars, 1862, *Daphnia longispina* O.F. Müller, 1776 and *Daphnia longiremis* G.O. Sars, 1861. Other microcrustaceans were represented by *Bosmina (Eubosmina) crassicornis* Lilljeborg, 1887, *Bosmina longirostris* (O.F. Müller, 1785), *Bosmina berolinensis* Imhof, 1888, *Chydorus sphaericus* (O.F. Müller, 1785), *Diaphanosoma brachyurum* (Lievin, 1848), *Leptodora kindtii* (Focke, 1844), *Eudiaptomus gracilis* (G.O. Sars, 1863), *Eudiaptomus graciloides* (Lilljeborg, 1888), *Heterocope appendiculata* Sars G.O., 1863, *Mesocyclops leuckarti* (Claus, 1857), *Thermocyclops oithonoides* (G.O. Sars, 1863), *Cyclops scutifer* Sars, 1863, and *Cyclops vicinus* (Sars, 1863). For more details, see Karpowicz and Ejsmont-Karabin (2017). *Eurytemora lacustris* was found in the cold water of the hypolimnion and metalimnion of all of the studied lakes (Fig. 2). It should be emphasised that before now, Lake Szurpiły was not a habitat for this species. The highest density (up to 8 ind. L^−1^) and biomass (up to 0.96 mg L^−1^) of *E. lacustris* was noted in Lake Wigry, in which it was a dominant species in the hypolimnion and metalimnion. The abundance and biomass of *E. lacustris* in lakes Hańcza and Szurpiły did not exceed 2 ind. L^−1^ and 0.15 mg L^−1^, respectively (Fig. 2c).

**Fig. 4:**
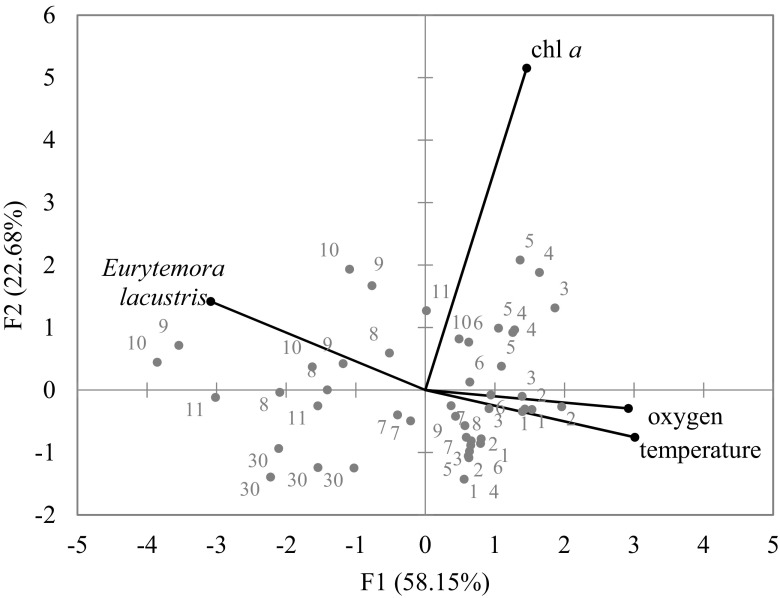
The relationships between the abundance of *Eurytemora lacustris* and the environmental variables (temperature, oxygen, and chlorophyll *a* – chl *a*) visualized by the principal component analysis (PCA). Grey dots and numbers represent the depth of sampling stations

**Fig. 5:**
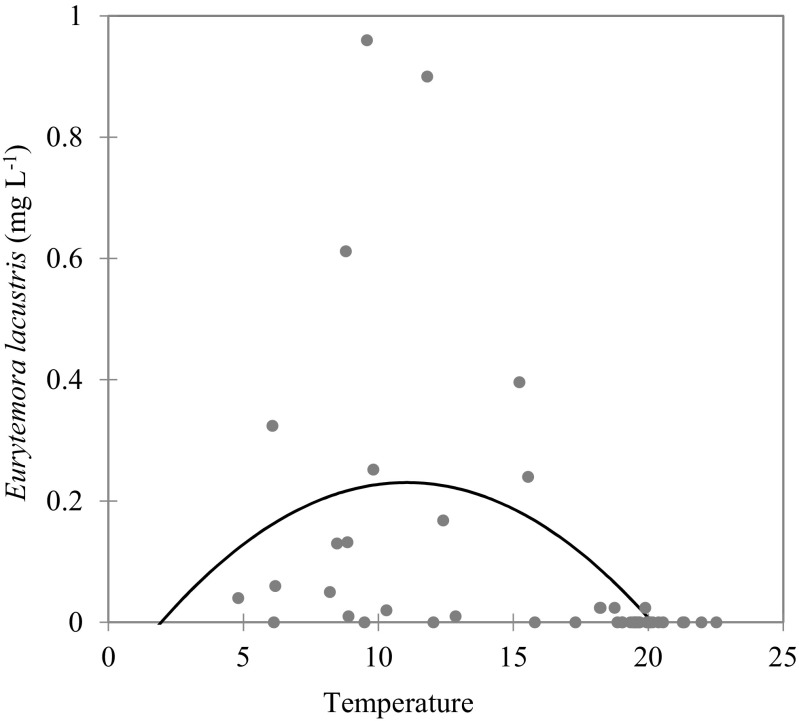
Nonlinear regression of *Eurytemora lacustris* biomass with temperature (R^2^ = 0.23). Grey dots are observations and the line is model

The vertical distribution of the *E. lacustris* population exhibited a clear heterogeneity with the maximal density in the thermocline of all of the studied lakes (Fig. 2c). In lakes Wigry and Hańcza, the abundance of *E. lacustris* was about 2–3 times higher in the metalimnion than in the hypolimnion. In Lake Szurpiły, *E. lacustris* was recorded exclusively in the thermocline. Statistical analysis showed that temperature of the water, oxygen concentration, and phytoplankton were important factors affecting the vertical distribution of *E. lacustris* (*F* = 6.83; *p* = 0.001). The contribution of each effect was evaluated by the Type I SS, and revealed that the most important factors were water temperature (*F* = 10.78; *p* = 0.002) and oxygen concentration (*F* = 8.56; *p* = 0.005). The results of the PCA analysis clearly divided environmental conditions into vertical profiles. The most epilimnetic stations were positively correlated with the first axis, while metalimnetic and hypolimnetic stations were negatively correlated with the first axis (Fig. 4). The horizontal axis (F1) was linked with temperature, oxygen concentration, and abundance of *E. lacustris*. The vertical axis (F2) was strongly linked with chlorophyll *a* concentrations (Table 2). The results of the PCA analysis indicated that *E. lacustris* preferred a low water temperature and could tolerate low oxygen concentration because of the higher phytoplankton concentrations (Fig. 4). The preferred temperature range of *E. lacustris* was between 6 and 16 °C. However, maximal densities of this species were observed at 9–11 °C (Fig. 5).

**Table 2:** Squared cosines table of the variables in the principal components analysis (PCA)

	F1	F2	F3	F4
*E. lacustris*	**0.757**	0.062	0.008	0.172
Temperature	**0.722**	0.018	0.168	0.092
Oxygen	**0.678**	0.003	0.295	0.024
Chl *a*	0.169	**0.824**	0.003	0.004

Values in bold correspond for each variable to the factor for which the squared cosine is the largest

**Fig. 6:**
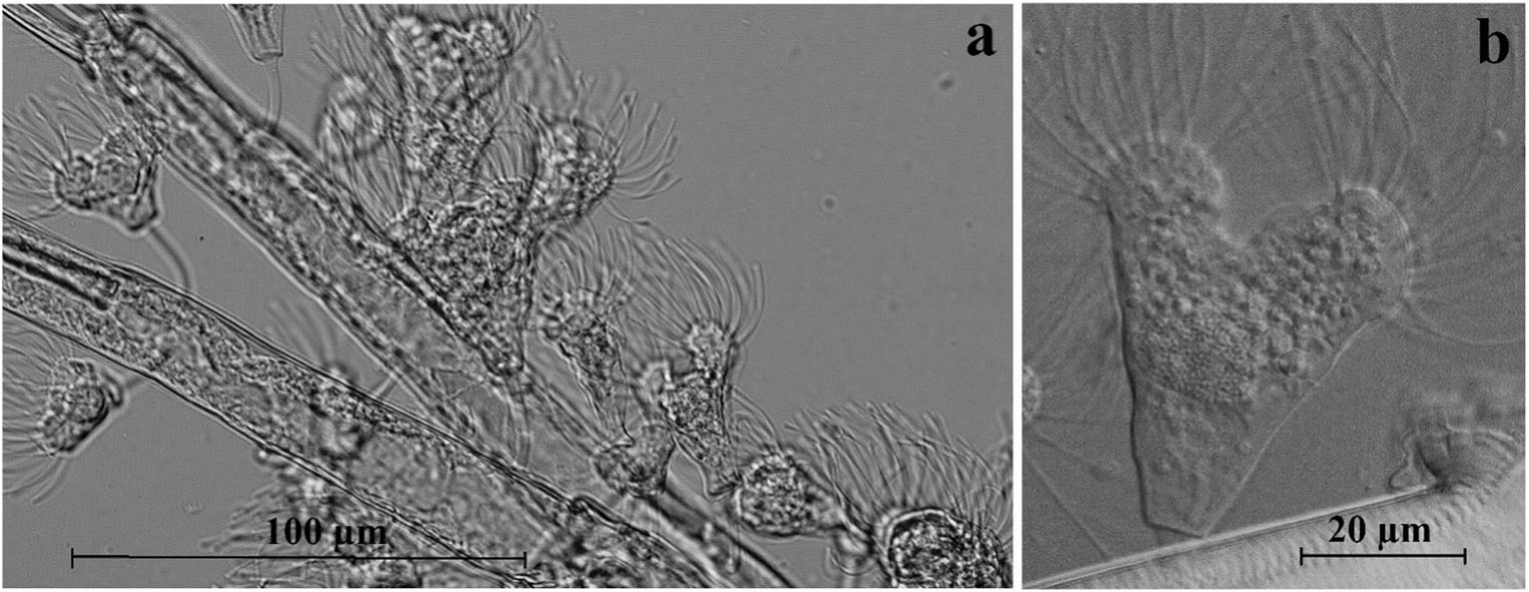
Epibionts on the furcal branches of *Eurytemora lacustris* (**a**), a single specimen of *Acineta tuberosa* (**b**) - Body (25–70 μm in size without stalk and tentacles) with two distinct protrusions, each bearing tentacles up to 55 μm in length. The stalk is two times shorter than the body (usually 12–30 μm long and 2.5–4 μm wide). Lorica, used for the characterisation of species and genera, is not visible, probably due to formalin fixation

A large part of the *E. lacustris* populations had epibiont ciliates, while all other species of zooplankton did not have epibionts. The prevalence of epibionts ranged from 33.3% in Lake Szurpiły to 50.0% in Lake Wigry (Table 3). The density of these epibionts varied in a very wide range from 5 to 112 individuals per specimen of *E. lacustris* (most often it was approximately 40–50 ind.). Up to 95% of all epibionts were attached to the abdomen and furca. These epibionts were difficult to identify but resembled the suctorian *Acineta tuberosa* (Pallas, 1766) Ehrenberg, 1833 presented in Foissner et al. (1995) by other authors. It should be noted that only one ciliate species was present on a single specimen of *E. lacustris*, but in different stages of the life cycle (Fig. 6).

**Table 3:** Quantifying epibiont numbers (± SD) in populations of *Eurytemora lacustris*

	Hańcza	Wigry	Szurpiły
Mean epibiont intensity	49.5 ± 10.2	38.7 ± 29.9	44.5 ± 10.6
Prevalence (%)	44.4	50.0	33.3
Host sample size (N)	9	34	6

## Discussion

We found the presence of *E. lacustris* in three mesotrophic, deep (>40 m) and well oxygenated (>4.5 mg O_2_ L^−1^) lakes. This may indicate the good ecological status of the lakes. In Lake Szurpiły, *E. lacustris* has never been recorded before. In the second half of the twentieth century, *E. lacustris* disappeared from Lake Wigry due to increasing eutrophication and anthropogenic pressure (Karabin and Ejsmont-Karabin 1999). After a significant reduction in phosphorus load from the catchment and biomanipulation at the end of the twentieth century, water quality radically improved in Lake Wigry (Kamiński 1999). As a result, an increase of the *E. lacustris* population in Lake Wigry was registered in 2007–2012 (Karpowicz and Górniak 2013). Similar results were found in other lakes, where re-oligotrophication is a well-documented phenomenon (Jeppesen et al. 2005), and *E. lacustris* has become more common in deep lakes of central Europe (Kasprzak et al. 2005; Maier et al. 2011). This makes *E. lacustris* an excellent indicator of the effective restoration of deep lakes in Europe. Our study showed that the abundance of *E. lacustris* in the deep water of Lake Wigry was around 8 ind. L^−1^, a value which is close to the winter maximum of this species (Błędzki and Rybak 2016). Literature data show that in the summer, this species is generally restricted to the cold hypolimnion, and the majority of its biomass is restricted to layers below the 10 °C isotherm (Weiler et al. 2003; Kasprzak et al. 2005). This study revealed the highest abundance of *E. lacustris* in thermocline zones. The preferred temperature range was between 6 and 16 °C, with an optimum from 9 to 11 °C. We have also found single individuals of *E. lacustris* in warm surface water.

The literature data indicated the large amplitude of diurnal vertical migrations of *E. lacustris*, but this amplitude clearly decreased from May to September. During the summer stagnation, the mean day depth and mean night depth were very similar (Kasprzak et al. 2005). However, the environmental factors which caused the relatively stable vertical distribution of *E. lacustris* in the summer are unclear. Besides the temperature and oxygen conditions, the light intensity is a factor responsible for their distribution (Kasprzak et al. 2005). Our results suggest that food resources may be an important factor in addition to water temperature and oxygen. We found that *E. lacustris* could temporarily tolerate warmer water and lower oxygen concentrations probably due to the higher availability of food resources. The food resources in the thermocline zone of lakes Hańcza and Wigry were much higher and better quality than in the hypolimnion. The hypolimnetic zones were strongly dominated by cyanobacteria, which are an inappropriate and poor quality food for zooplankton (e.g. Kosiba et al. 2017). Freshwater calanoids possess numerous chemoreceptors on their antennae and mouthparts that are sensitive to the toxins produced by cyanobacteria and feed selectively by choosing particles of higher quality (DeMott 1986). Kasprzak et al. (2005) suggested that insufficient food supply in combination with low food quality may restrict the occurrence of *E. lacustris* in some cases. The food resources in the thermocline zone of lakes Hańcza and Wigry were much higher and better quality than in the hypolimnion. Studies by Vezhnovets et al. (2012) on the stomach content of calanoid copepods, examined by scanning electron microscopy, showed that the food pellet of *E. lacustris* mostly contained small (<40 μm in length) pennate diatoms, that were damaged and difficult to identify. The authors underlined that the dominant algal group/species in lake is the main and easily available food resource for *E. lacustris*. In turn, the lack of *E. lacustris* in the hypolimnion of Lake Szurpiły could be the result of the worse aerobic conditions.

Our study revealed that a large part of the *E. lacustris* population had epibiont ciliates, in contrast to other species of zooplankton. Several studies have reported the presence of epibionts on various species of calanoid copepods (Turner et al. 1979; Nagasawa 1986; Valbonesi and Guglielmo 1988; Chiavelli et al. 1993; Green and Shiel 2000; Utz and Coats 2005, 2008; Visse 2007). Peritrich ciliates, such as *Vorticella* spp. and *Zoothamnium* spp. have been found on copepods in the Gulf of Gdańsk (Wiktor and Krajewska-Sołtys 1994). Utz and Coats (2005) found that the common epibiont of estuarine *Eurytemora affinis* Poppe, 1880 (Chesapeake Bay, USA) was the sessile peritrich ciliate *Zoothamnium intermedium* Precht, 1935. We have found that relic *E. lacustris* in lakes of north-eastern Poland had suctorian epibiont ciliates, namely *Acineta tuberosa* Ehrenberg, 1833. This phenomenon may have not only a local character. It seems that the same suctorian species were illustrated, but not identified and described, on *E. lacustris* from Ratzeburger Lake complex in Germany (Maier et al. 2011 - Fig. 1b). The epibiont prevalence has generally been observed at the time when the host species are very abundant and dominate plankton communities (Hirche 1974; Chiavelli et al. 1993). The results of this study indicated a strong preference of epibiont ciliates for *E. lacustris* despite their low share in zooplankton communities.

Epibiosis is essentially a commensal relationship, although new data suggest that epibionts can have negative effects on some life-cycle traits of the Calanoida host (Visse 2007; Souissi et al. 2013). A laboratory experiment with epibionts attached to the calanoid copepod *Acartia bifilosa* (Giesbrecht, 1881) (Gulf of Riga, Estonia) suggested that animals infested with epibionts were less viable than the non-infested animals. Another laboratory experiment, using 2D infrared video techniques to observe the behavior of heavily infested *E. affinis*, revealed that epibionts could negatively affect the behavior of the host, in terms of swimming activity, e.g. break, cruise, sink, and jump (Souissi et al. 2013). The high proportion of infestation also dramatically affected the mating success of *E. lacustris* in laboratory conditions (Souissi et al. 2013). The improvement in water quality has led to an increase in the population of *E. lacustris* in deep lakes; however, epibiont ciliates could be a threat for *E. lacustris* populations and may play a substantial role in determining community production and dynamics. Our results showed that *E. lacustris*, with the mean biomass of 75 μg, had on its body “epibiotic luggage” with a biomass of about 1 μg, accounting for 1.3% of its biomass. This luggage appears to be small but may slow down the movement of *E. lacustris* and its vertical distribution. However, this assumption requires further studies. It is also necessary to address another question - why do suctorian ciliates colonize only one (in this case less numerous *E. lacustris*) among many other (more numerous) zooplankton species?

## Conclusions

The results of this study revealed the highest abundance of *E. lacustris* in thermocline zones and indicated that this species could temporarily tolerate warmer water and lower oxygen concentrations probably because of the better food resources. A large part of the *E. lacustris* population had the epibiont ciliate *Acineta tuberosa*, while other species of zooplankton did not have suctorian epibiont ciliates. This phenomenon may not be only a local character. Few laboratory experiments on *E.* species revealed that epibionts could negatively affect the behavior of the host and dramatically affected the mating success. Nowadays, the improvement in water quality has led to an increase in the population of *E. lacustris* in deep lakes. However, epibiont ciliates could be a threat for *E. lacustris* populations and may play a substantial role in the production, distribution, and community dynamics.
